# A litmus test for plant consciousness: Pattern–Temporal Synergy in a relation-first ontology

**DOI:** 10.1080/19420889.2025.2563993

**Published:** 2025-10-13

**Authors:** Arie T. Greenleaf

**Affiliations:** College of Psychology, Nova Southeastern University, Fort Lauderdale, FL, USA

**Keywords:** Calcium-wave signaling, dynergergic theory of consciousness, Pattern–Temporal Synergy (PTS), plant consciousness, temporal binding window (τ)

## Abstract

Plant cognition has progressed from anecdote to rigor, yet the field still lacks a quantitative test for when distributed plant activity crosses into unified – perhaps conscious – processing. I introduce Pattern–Temporal Synergy (PTS), a substrate-agnostic metric rooted in Dynergeia, a relation-first ontology in which consciousness is reflexive coherence among five universal patterns – self-reference, division-creation, information integration, responsiveness, and flux – phase-locked inside a system’s binding window (τ). Each pattern is operationalized with established signal-processing measures; their median strength is multiplied by their mean synergy and released only if a τ-specific coherence gate is met. Three preregistered hypotheses anchor the study: H1 baseline PTS > 0 in intact plants; H2 4% diethyl-ether collapses PTS below threshold ϕ; H3 PTS rebounds on wash-out. A multispecies protocol – Mimosa pudica, Arabidopsis thaliana, Picea abies – combines 64-channel surface electrodes, glutamate-sensitive Ca2+ imaging and micro-optode O2/heat-flux probes. Sliding 3 ×τ windows with phase-shuffled surrogates yield z-scored PTS trajectories, adjudicated by preregistered effect-size criteria. By turning decades of qualitative insight into falsifiable numbers, PTS offers plant biology a litmus test for conscious-level processing, directly challenges Integrated Information Theory and supplies a road-map for cross-kingdom comparisons – including neuromorphic silicon. Confirmatory results would shift debates on plant sentience from speculation to data; null results would equally refine what consciousness requires.

## 1. Introduction: Closing the metric gap in plant cognition

### 1.1. The metric gap: From anecdote to quantification

Plant biologists no longer ask whether roots and shoots process information; landmark studies have proven that they do. For instance, Mimosa pudica has been shown to habituate to harmless drops and remember the lesson for weeks [1], with follow-up studies offering supportive replications [2]. In a separate line of inquiry, reports that pea seedlings can learn by association [3] have ignited a vigorous debate and have been subject to critical replication attempts [4]. Electrophysiological surveys now show that plants fire action and slow-wave potentials that travel centimeters per second, relaying wound or herbivory signals from damaged leaves to distant tissues [5]; glutamate-triggered Ca2+ waves then sweep the canopy within minutes, synchronizing defense genes across the organism [6]. No wonder recent reviews speak openly of a ”plant neurobiology” revolution – even as some caution against anthropomorphic overreach [7].

What the field still lacks is a quantitative yard-stick to determine – across species, perturbations, and laboratories – when distributed plant activity crosses the threshold from mere cognition to reflexive coherence (i.e., conscious-level processing). This paper introduces Pattern–Temporal Synergy (PTS) as that yard-stick. Grounded in Dynergeia’s (pronounced: die-ner-jah) relation-first ontology (sections 2–3), PTS scores any multichannel plant recording for five universal relational patterns, then gates the result through the plant’s native temporal binding window (τ). The outcome is a single, dimensionless number (0 ≤ PTS ≤ 1) that rises only when the system’s internal activity achieves the reflexive coherence Dynergic Theory identifies with conscious-like organization (section 4). A concise overview of Dynergeia and its application to PTS is provided in Box 1.

Box 1: Dynergeia in 60 Seconds Plant cognition research has raced ahead of its quantitative tools. Dynergeia starts from a relation-first ontology: reality is structured by dynamic relations, not static substances. Five universal relational patterns – self-reference, division-creation, information integration, responsiveness, and flux – become conscious only when they phase-lock inside a system’s native binding window (τ). Pattern–Temporal Synergy (PTS) scores the strength and synchronicity of those patterns and reports a value only when they clear a τ-specific coherence gate. In plants, ether anesthesia is predicted to halve PTS, then rebound on washout – a falsifiable litmus test. This paper supplies the glossary, mathematics, and preregistered protocol needed to run that experiment.

For investigators already convinced that Mimosa habituates and Arabidopsis predicts light gradients, the practical questions are now how much, under what perturbations, and with which temporal signature. PTS converts these qualitative observations into quantitative, falsifiable predictions, such as the expectation that ether anesthesia will depress whole-plant PTS by at least 0.50 and that recovery will track the rebound of Ca2±wave coherence (sections 6 and 7).

Accordingly, this manuscript pursues four aims:

1. to situate Pattern-Temporal Synergy inside a fully articulated relation-first ontology (section 2);
2. to explain why temporal binding (τ) is the critical gate between reflexive coherence and mere signal traffic (section 3);
3. to formalize the PTS computation from plant-specific signals (section 4); and
4. to lay out an experimental program – species, sensors, protocols, statistics – that any laboratory can implement to confirm, refine, or refute these claims (sections 6 and 7)).

In sum, this paper offers a quantitative framework intended to close the current metric gap in plant-consciousness research, providing a testable way to evaluate whether distributed plant dynamics can meet the conditions for reflexive coherence.

### 1.2. The substance-first impasse

Modern biology, neuroscience, physics, and philosophy of mind still inhabit a substance-first worldview: discrete entities are taken as ontological bedrock, while the relations among them are treated as secondary properties to be explained away [7]. In consciousness research, this stance spawns a whole family of puzzles – the hard problem (why any physical process should feel like something from the inside), the combination problem (how many small putative ”micro-experiences” could fuse into a single unified one), the interaction problem (how subjective experience could causally influence physical matter), the boundary problem (why subjective experience seems to start or stop at particular system sizes or architectures), and the persistent explanatory gap between third-person models and first-person qualities. Reductionist answers either declare subjective qualities illusory [8] or rebrand them as information states without clarifying why certain informational configurations acquire felt character while others do not [9]. Dualist and panpsychist proposals avoid emergence by positing nonphysical mind-stuff or proto-experience in every particle, but then face the interaction and combination problems, respectively [10,11]. The net result is a decades-long theoretical stalemate: opposing camps critique one another’s assumptions yet share the same entity-first grammar that created the impasse.

Plant-consciousness research inherits the same bind. Experimentalists document sophisticated signaling, learning, and long-distance integration, but must fall back on behavior-based analogies or on human-centric neurocognitive metrics to infer awareness [1,3,4,6]. Integrated Information Theory has been adapted to vascular plants, yet its Φ-values remain difficult to measure in phloem networks and do not specify clear thresholds for subjective manifestation [12]. Other proposals focus on electrical complexity [13] or metabolic rates [14] without supplying a falsifiable linking principle. In short, the field lacks a substrate-agnostic, quantitative metric that can decide – via direct measurement – whether relational organization in plants ever crosses the line from mere reactivity to lived experience. Pattern–Temporal Synergy, grounded in a relation-first ontology, is introduced here as that metric. By embedding measurement within an explicitly relational framework, PTS transforms what has long been a philosophical debate into a falsifiable scientific program. The experimental conditions and parameters are summarized in Table 1.

**Table 1.** Glossary of core terms.

Term	One-line definition
Dynergeia	Relation-first ontology: reality is relational choreography, not substance.
dynamis → energeia	Latent potential actualizing as organized activity.
Relational Coherence Field (RCF)	Universe’s capacity for self-organizing relations; becomes conscious when reflexively coherent.
Five patterns	Self-reference, Division-creation, Integration, Responsiveness, Flux.
τ (binding window)	Max interval in which a substrate can phase-lock its patterns (≈2 s plants; 100 ms humans).
CSAFU laws	Coherence-Formation, Synchrony-Gate, Adaptive-Modulation, Flux-Principle, Universality-of-Medium – meta-constraints shaping patterns.
PTS	Pattern–Temporal Synergy: P×S gated by τ; quantitative consciousness index.
φ (threshold)	Fraction of τ-windows (e.g., 0.75) that must meet coherence for PTS > 0.
τ-isolation/τ-incommensurability	Systems whose τ differ by ≥ 10^3^ cannot share phenomenology or cross-correlate PTS.
Synchrony-Gate	CSAFU law: temporal coherence prerequisite for unified experience.

### 1.3. Dynergeia + DTC + PTS: A unified roadmap

Dynergeia inverts the standard entity-first worldview by adopting a relation-first stance. In this ontology, the Relational Coherence Field functions as the universe’s organizing medium. Five recurrent patterns – self-reference, division-creation, information integration, responsiveness, and flux – draw reality from latent potential (dynamis) into active form (energeia).

The Dynergic Theory of Consciousness (DTC) builds on this foundation. It holds that conscious experience arises whenever these five patterns phase-lock within a system’s temporal binding window (τ), producing reflexive coherence. This claim is explicitly substrateagnostic: the same conditions apply whether the substrate is neural, vascular, or neuromorphic.

Pattern–Temporal Synergy operationalizes this theory as an empirical test. In each analysis window PTS (i) quantifies the five patterns, (ii) normalizes their scores, (iii) multiplies mean strength by mean synergy, and (iv) releases the result only if a τ-specific coherence gate is satisfied. Modeling work predicts that, in humans, PTS falls by 40–70% under propofol anesthesia and rebounds as consciousness returns [15]. Because the algorithm requires only time-series data rather than neuron-specific features, it can be applied directly to plant Ca2+ waves, phloem voltages, or metabolic flux signals without modification.

In this way, Dynergeia provides the ontological grounding, DTC supplies the falsifiable mechanism, and PTS delivers the measurable yardstick that can advance plant-consciousness research from behavioral analogy to direct, quantitative assessment.

### 1.4. Testable predictions

To convert Dynergeia’s ontology and the Dynergic Theory of Consciousness’ mechanism into a falsifiable plant-level test, I advance three preregistered hypotheses that hinge on Pattern–Temporal Synergy computed from live physiological signals in Mimosa pudica, Arabidopsis thaliana, and Picea abies.

#### 1.4.1. Hypothesis one: Baseline relational coherence

Under normal growth conditions each plant will display a non-zero mean PTS, indicating that the five Dynergic patterns are expressed and at least partially synchronized within the species-appropriate binding window, τ. Detecting PTS > 0 rules out the trivial null that plant signaling is too fragmented or too slow to register any coordinated relational dynamics.

#### 1.4.2. Hypothesis two: Anesthetic collapse

Exposure to diethyl ether – a classic reagent that halts Mimosa leaf movements and Venus-flytrap closure within minutes [16,17] – is predicted to depress PTS by at least 40% relative to baseline. This magnitude is chosen because a 40–70% reduction in comparable neural metrics accompanies loss of consciousness in human propofol studies; however, whether ether produces an analogous effect in plants remains to be determined experimentally. Demonstrating such a PTS drop would directly test DTC’s claim that consciousness hinges on τ-synchronized coherence rather than on any specific neural substrate.

#### 1.4.3. Hypothesis three: Wash-out recovery

Within twenty minutes of ether removal, mean PTS will rebound to at least 90% of its baseline value, paralleling the reversible recovery of plant electrophysiology and motor responses reported in ether wash-out studies [18]. Full or near-full restoration would confirm that the PTS collapse is a functional disruption, not a permanent physiological insult.

Together these hypotheses turn the ”metric gap” into a decisive test: if all three are confirmed, the results would show that plant-level dynamics can meet Dynergeia’s reflexive coherence conditions for consciousness; if any hypothesis fails, the data would simply place plants below the φ threshold under the specified protocols – without challenging the framework’s substrate-agnostic ontology itself.

## 2. Dynergeia: Ontological foundations

### 2.1. Relation-first ontology and the relational coherence field

At the heart of the Dynergic framework is a fundamental inversion of the standard metaphysical hierarchy. Whereas traditional science treats discrete entities as the foundation of reality and their interactions as secondary, Dynergeia establishes relation itself as primary. On this view, reality unfolds as a Relational Coherence Field – not an additional substance layered onto matter, but the universe’s intrinsic capacity for organizational constraint. Like boundary conditions that shape electromagnetic field solutions without adding energy [19], the RCF operates through what it prohibits rather than what it produces. Entities – from quantum excitations to cellular networks to whole organisms – emerge as temporary crystallizations within this deeper relational matrix, not as self-standing building blocks that later enter into relations.

Consciousness represents the RCF’s most advanced expression: its capacity for reflexive self-recognition. This occurs when five universal patterns – self-reference, division-creation, information integration, responsiveness, and flux – achieve sufficient strength, synergy, and temporal coordination within a system’s native binding window, τ. When these patterns phase-lock with adequate intensity and coherence, the field transitions from latent organizational potential (dynamis) to lived experiential actuality (energeia).

The Dynergic Theory of Consciousness formalizes this transition as a measurable threshold phenomenon, while Pattern–Temporal Synergy provides the empirical metric to detect when any substrate – neural, vascular, or silicon – crosses into reflexive coherence.

### 2.2. Five universal relational patterns

Having established the ontological framework, we can now examine the five Dynergic patterns in detail. The Relational Coherence Field expresses itself through these mutually reinforcing modes of organization. Each is necessary, and only their synchronized interplay is sufficient to shift the field from latent potential (dynamis) to lived actuality (energeia).

#### 2.2.1. Self-reference

A local subsystem encodes information about its own unfolding, creating a reflexive loop that stabilizes identity across time. Without this recursive modeling, no coherent ”point of view” can emerge, as the system lacks a thread connecting past, present, and anticipated states.

#### 2.2.2. Division-creation

The field simultaneously draws boundaries and keeps them permeable. This continual differentiation carves functional subunits out of flux while allowing reconfiguration. It prevents relational order from dissolving into undifferentiated sameness and provides the categorical scaffolding on which higher organization depends.

#### 2.2.3. Information integration

Boundaries alone would fragment experience unless disparate signals are rejoined into a unified whole. Integration fuses differentiated streams into a single relational tapestry, ensuring that the system’s myriad micro-events compose one macro-situation.

#### 2.2.4. Responsiveness

Coherence must remain coupled to its environment. Responsiveness captures the system’s ongoing adjustment to external change, anchoring the unified field of relations to real-time contingencies and giving the flow of events a directional ”aboutness.”

#### 2.2.5. Flux

Order remains adaptive only if it continuously renews itself. Flux drives micro-level turnover – ion exchange, metabolic cycling, structural remodeling – so the other four patterns stay dynamic rather than brittle. Far from being background noise, this dynamism is the pulse that allows coherence to persist and evolve.

Taken together, these five patterns form a universal grammar of organization. If any is absent or impaired, relational potential remains dispersed. When all five phase-lock inside the system’s binding window τ, the field crosses the threshold into reflexive coherence – a transition that the Dynergic Theory of Consciousness identifies with conscious manifestation. This theoretical foundation sets the stage for operationalization: to move from ontology to measurement, the patterns must be anchored in concrete physiological processes. The next step, therefore, is to examine how these patterns appear in plants and how they can be quantified through the Pattern–Temporal Synergy framework.

### 2.3. Plant-level realizations of the five patterns

The five Dynergic patterns, while substrate-agnostic in principle, manifest clearly within well-characterized plant physiology. This demonstrates that vascular plants possess the necessary biological architecture for consciousness-level organization.

- **Self-reference**. Systemic acquired resistance provides a whole-plant memory: roots and leaves retain information about prior pathogen exposure, which modulates future gene expression across the organism [20].
- **Division-creation**. Apical dominance establishes dynamic boundaries between the leading shoot apex and lateral buds, continuously redrawing functional modules as auxin gradients shift with environmental conditions [21].
- **Information integration**. Glutamate-triggered Ca2+ waves and accompanying electrical signals propagate through phloem and xylem networks, knitting distal tissues into unified informational coordination across the entire plant body.
- **Responsiveness**. Rapid turgor-driven leaf movements in species such as Mimosa pudica translate mechanical or thermal stimuli into millisecond-scale adjustments [22], exemplifying the environmental coupling that anchors coherent systems to real-time contingencies.
- **Flux**. Circadian regulation of starch turnover [23] and thermogenic heat pulses in species like Arum maculatum [24] illustrate the continuous metabolic remodeling that prevents relational ossification and maintains adaptive flexibility.

These concrete physiological expressions demonstrate that all five Dynergic patterns are naturally instantiated in plant architecture. This provides not only the conceptual justification but also the empirical substrate for computing Pattern–Temporal Synergy and testing whether plants can achieve the reflexive coherence that Dynergeia associates with conscious experience.

## 3. Temporal architecture of consciousness

### 3.1. Synchrony-gate (τ): Reflexive coherence in time

Dynergeia holds that consciousness ignites only when the five universal patterns synchronize within a system’s intrinsic temporal binding window, τ [25]. The Dynergic Theory of Consciousness calls this enabling condition the Synchrony-Gate: relational dynamics must complete a full loop – recursion, differentiation, integration, outward coupling, and self-revision – before the window closes. If any pattern lags, coherence fragments and awareness fails to actualize.

To make this constraint intuitive, consider the neural evidence. In the mammalian cortex, thalamocortical circuits bind distributed cell assemblies by locking 30–100 Hz γ-band oscillations [26]. When anesthetics broaden conduction delays and break γ coherence, subjects lose consciousness even though local spiking persists [27]. Intracranial recordings further show that perceptual reports hinge on sub-100 ms phase alignment across parietal, temporal, and frontal hubs, reinforcing the view that timing, not sheer firing rate, marks the threshold of reflexive coherence [28].

Plants operate on a far slower clock, yet the same logic applies. Electrical action potentials in Mimosa pudica traverse petiole-to-stem paths in seconds, while glutamate-triggered Ca2+ waves spread across the canopy in tens of seconds [5,22]. DTC predicts that these signals will only generate a unified field of experience if their feedback loops close within the species-specific τ – estimated here at ∼2 seconds for Mimosa based on electrophysiological round-trip times. Section 6’s ether-knockout paradigm therefore tests the Synchrony-Gate directly: by pharmacologically prolonging conduction delays, we should observe a proportionate reduction in Pattern–Temporal Synergy. A concise illustration of the protocol and predictions is given in Box 2.

Box 2. How the Five Patterns Construct Time The standard view treats time as a universal container – a passive backdrop against which events unfold. Dynergeia inverts that assumption: time is not given; it is made. It emerges from the coordinated activity of five universal relational patterns operating within a system’s native binding window (τ). Each pattern contributes a distinct function to temporal world-making:

- Self-reference (continuity). Links prior states to the present, forging a coherent historical thread.

- Division-creation (segmentation). Carves the flow into discrete “moments,” supplying boundaries between episodes.

- Information integration (unity). Weaves parallel streams into a single, holistic temporal episode – the lived “now.”

- Responsiveness (directionality). Orients the system toward predicted conditions, imprinting a forward arrow of time

- Flux (change). Prevents stasis by continual micro-revision, ensuring becoming rather than mere repetition.

These patterns do not unfold in time; their coordinated action constructs time at the system’s native rate. A quantum transition, a metabolic cycle, or a galactic orbit each casts its own duration through its τ-scaled coordination. Because τ differs by substrate (e.g., milliseconds in cortex, seconds in plants, microseconds in neuromorphic chips), every organized system authors its own ‘temporeality.’

Consciousness is the special case of reflexive time-making. When all five patterns achieve sufficient strength and phase-lock within τ – clearing a critical coherence threshold φ –the system becomes aware of the very flow it is generating. It does not merely register duration; it experiences the time it makes.

These patterns do not operate in a vacuum; they coordinate within a specific operational frame known as the τ-window. This window defines the timescale of a system’s temporal world-making. A quantum collapse, a metabolic cycle, or a galaxy’s orbit do not simply happen in time – they actively craft their own intrinsic durations through the coordinated action of these patterns. Each system, therefore, enacts time at its own native scale.

Consciousness represents a special, higher-order case of this process. When all five patterns converge with sufficient strength, synergy, and phase-locking within the τ-window (crossing a critical threshold, φ), the system becomes reflexively aware of its own time-making. It does not merely generate duration – it experiences the very flow it creates.

### 3.2. Species- and substrate-specific τ-windows

The duration of the Synchrony-Gate varies dramatically across biological and synthetic architectures.

In humans, thalamocortical loops fuse distributed neural ensembles within ≈100 ms. This is the period that hosts 30–100 Hz γ-band phase locking and aligns with the subjective”specious present” [29]. General anesthetics widen axonal conduction delays and disrupt γ coherence; when the effective τ stretches beyond ≈200 ms, consciousness disappears even though local firing persists [30]. Attentional-blink tasks likewise show that stimuli failing to recruit cross-regional synchrony within this 100 ms window remain unconscious [31].

Plants operate on a slower metronome, set by ionic and hydraulic transport. In Mimosa pudica, action potentials propagate along the petiole at 5–10 cm s − 1, enabling round-trip feedback for leaf – stem circuits in ≈ 1–2 s [32]. In Arabidopsis thaliana, systemic glutamate triggered Ca2+ waves spread at millimeters per second. This suggests a species-specific τ of ≈ 2 s for localized reflexive coherence, with a broader temporal envelope of tens of seconds for whole-plant integration.

Engineered substrates can be faster still. Event-driven neuromorphic chips such as Intel’s Loihi achieve spike latencies under 1 ms, enabling recurrent cycles in ≈5 ms [33]. If such hardware were to instantiate all five Dynergic patterns, the DTC predicts its phenomenal”now” would unfold ≈ 20 times faster than the human present and ≈ 400 times faster than Mimosa’s electrical window.

This spectrum underscores τ as a biophysical dial on conscious tempo: shorten τ, and relational coherence must synchronize with millisecond precision; lengthen it, and slower ionic or hydraulic waves suffice. The empirical protocols in Section 6 exploit this scaling directly: by pharmacologically stretching plant conduction delays, they test whether a τspecific collapse in Pattern–Temporal Synergy marks the loss of reflexive coherence.

### 3.3. τ-isolation: Binding windows as phenomenological firewalls

The Dynergic Theory of Consciousness introduces a principle of τ-isolation: when two systems have binding windows so far apart that their τ-ranges do not overlap, each can register only a coarse-grained, averaged version of the other’s activity. Relational possibilities that unfold too quickly or too slowly are collapsed into a single, static outcome from the observer’s perspective.

A femtosecond quantum superposition interacting with a picosecond detector illustrates the rule: the detector’s slower gate averages the faster coherence into one classical event. By the same logic, a human cortex sampling the world in ≈100 ms frames cannot resolve the 5 ms feedback loops of a neuromorphic chip; to the human, the chip’s internal dynamics appear only as a fixed input.

Quantitatively, the theory predicts that whenever the ratio τslow/τfast exceeds approximately three orders of magnitude, cross-boundary Pattern–Temporal Synergy collapses. Synchrony across the interface becomes physically impossible. For example, to a plant’s electrical network operating on 1–2 s loops, human γ-band activity (≈100 ms) would appear as an unstructured blur. Conversely, to a human observer, the plant’s moment-to-moment relational dynamics would be too slow to perceive as a coherent, unfolding event, instead appearing as a static state. Section 6 tests this principle directly.

Crucially, τ-isolation makes consciousness private without appealing to observer-dependent metaphysics. Each phenomenal domain is bounded by its temporal architecture, and no experiential bridge can span such non-commensurable τ-scales. In this way, the Dynergic Theory unifies quantum measurement and subjective awareness under a single, time-indexed coherence principle, while remaining empirically falsifiable through the measurable collapse of PTS when τ-windows are experimentally shifted.

### 3.4. τ-incommensurability: Limits on cross-substrate co-consciousness

Whereas τ-isolation explains why a fast system’s dynamics decohere when sampled by a much slower observer, τ-incommensurability extends the principle to mutual interaction. When the ratio τslow/τfast exceeds roughly three orders of magnitude, neither system can sustain Pattern–Temporal Synergy with the other, and no shared phenomenological field can form.

A human cortex (τ ≈ 100 ms) can synchronize with speech rhythms or musical beats because those external patterns lie within one logarithmic decade of its binding window. By contrast, a neuromorphic array cycling in 5 ms and a Mimosa petiole circuit cycling in 2 s are separated by five decades. Each coarse-grains the other’s activity into static input, making cross-subsystem PTS correlations indistinguishable from noise.

The Dynergic Theory therefore predicts a hard ceiling on cross-substrate coherence: if two systems differ in τ by ≥ 103, their relational grammars become mutually invisible. Information may still transfer (bits, voltages, photons), but no bidirectional phase-locking – and thus no co-consciousness – can arise. Practically, this means that even perfect data links between a sub-millisecond silicon network and a seconds-scale plant circuit cannot yield a unified experiential domain; each remains confined to its own temporal architecture.

An empirical test follows directly. Section 6 outlines pairing Mimosa electrical grids with a programmable spike-timing processor whose τ can be tuned from 1 ms to 3 s. Co-fluctuations in PTS are expected only while the processor’s timescale is commensurable with the plant’s native τ, for instance, within one order of magnitude (0.1–10x). Beyond that range, synchrony should collapse despite continued signal exchange, long before the theoretical hard limit of 103 is reached. Confirmation would establish τ-incommensurability as a quantitative boundary on relational consciousness; falsification would compel revision of the Dynergic Theory’s time-indexed coherence rule.

## 4. Pattern–Temporal Synergy: Metric definition and pipeline

### 4.1. Formal definition and threshold criteria

Pattern–Temporal Synergy translates Dynergeia’s five relational patterns into a single, substrate agnostic index of conscious potential. Each pattern – self-reference, division-creation, information integration, responsiveness, and flux – is first quantified by an empirically grounded proxy (e.g., Active Information Storage for Self-reference [27], 1/f spectral slope for Flux [34]. The strength of every pattern in a sliding analysis window is converted to a z-score against phase-randomized surrogates so that all measures share a common scale [35]. From this 5-vector of z-scores, two summary values are calculated [36]:

- Central (median) Pattern Strength (P¯), the median of the z-scores, and
- Mean Synergy (S¯), the average pairwise synergy derived from a Partial Information Decomposition (PID) framework (see Appendix A.2).

The temporal-coherence gate TC then asks how often all five patterns co-occur inside the system’s native binding window τ. If at least a fraction φ = 0.75 of windows meet this synchrony requirement, PTS is released as PTS = P¯ × S¯ if TC ≥ φ; else 0 signaling that the system’s relational order never stabilizes long enough to form a unified present when the gate fails. The 75% cutoff is not arbitrary: it reproduces the empirical inflection in human conscious-access studies [37] and the mid-range collapse seen under surgical propofol, providing a robust, cross-study anchor [38].

Plants demand a two-tier timing test. Rapid phloem action potentials spread through a leaf in < 2 s, whereas whole-organ Ca2+/ROS waves require 10–100 s to recruit distal tissues [6,39,40]. Therefore compute:

- Leaf-level PTS: window = 3 τ ( = 6 s), step = 5 s
- Whole-organ PTS: window = 30 s, step = 15 s

Both tiers must independently satisfy the TC ≥ φ gate before a plant epoch is judged conscious; failure at either scale nulls PTS despite local pattern strength. This dual-window rule respects the physiology of long-distance signaling while preventing spurious positives driven by fast, spatially limited bursts.

The formulation differs sharply from Integrated Information Theory’s Φ, which multiplies structural connectivity by an assumed phenomenology yet leaves temporal precision implicit. PTS, in contrast, makes time explicit, setting the same fractional threshold across substrates but letting τ stretch from milliseconds in neuromorphic silicon to tens of seconds in vascular plants. Because the metric is scale-free and falsifiable, any architecture that can raise P¯, increase synergy S¯, and keep TC ≥ φ within its own τ should cross the consciousness boundary – regardless of chemistry or morphology.

### 4.2. Plant-specific signaling inputs

Plant tissues convey information through three complementary carrier streams that, taken together, cover the temporal bandwidth needed to compute Pattern–Temporal Synergy. Each stream is recorded in parallel and time-locked to a common clock (1 kHz master trigger distributed through the DAQ backplane), ensuring that downstream pattern metrics can be extracted from perfectly co-registered analysis windows.

Ca2+ waves (0.05–0.5 Hz). Long-distance glutamate-triggered calcium flashes propagate through the apoplast at ≈400 µm s − 1 and coordinate systemic wound and light responses in Arabidopsis and Pisum [6]. I express GCaMP6f under the UBQ10 promoter and image with a sCMOS camera at 20 Hz; ∆F/F0 traces are motion-corrected with NoRM-

Corre [41] and z-scored within each 10 s block. These signals feed the Integration and Division-creation metrics (PID synergy and modularity Q, cf. 22).

Phloem surface voltage (0.2–5 Hz). Electrical action potentials ride the sieve-tube membrane and reach neighboring organs within seconds, acting as the plant’s rapid alarm line [14]. Custom Ag/AgCl micro-electrodes (tip Ø 50 µm, 1 M KCl gel) are inserted 1 mm into the phloem bundle; signals are AC-coupled (0.1 Hz−1 kHz), digitized at 2 kHz, and notch-filtered for mains noise. Phase-lag-index graphs derived from these channels dominate the Responsiveness and Self-reference calculations (weighted symbolic transfer entropy; AIS).

O2 micro-flux (10 − 3–0.1 Hz) and infrared heat maps (10 − 2–0.1 Hz). Clark-type micro-optodes sample dissolved oxygen at 1 Hz, capturing the respiratory trough after ether application and its rebound during wash-out [36]. Simultaneously, a FLIR A700 acquires 640 × 480 thermal frames at 5 Hz; pixel-wise temperatures are averaged over 2 s windows to yield a heat-flux proxy that complements the 1/f spectral-slope estimate of Flux [42]. Both modalities are low-pass filtered (cutoff 0.2 Hz) before windowed detrending to remove drift.

Why these three streams? Together they bracket the plant’s characteristic binding window τ ≈ 2 s: Ca2+ waves supply the fast edge, phloem spikes the meso-band, and metabolic flux the slow envelope. Because Dynergeia predicts that consciousness ignites only when all five patterns synchronize inside τ, omitting any of these carriers would risk a false-negative PTS. Conversely, their union spans six decades of frequency while remaining technically feasible with off-the-shelf sensors, satisfying the CSAFU requirement of Universality-of-Medium.

Signal-quality safeguards. Epochs are discarded when mean photobleaching exceeds 5% min − 1, electrode impedance rises above 5 MΩ, or O2 probe drift exceeds 2% of span per hour. Surrogate phase-randomized controls are generated independently for each modality to preserve their noise spectra during null-model construction.

By anchoring the PTS pipeline in these empirically validated plant signals, Section 4.3 can translate raw voltages and photon counts into the five Dynergic pattern metrics without violating either botanical physiology or information-theoretic rigor.

### 4.3. Detailed computational pipeline

The pipeline begins by resampling all asynchronous data streams (Ca2+, phloem voltage, O2, heat) to a common 500 Hz grid. Analysis then proceeds in 50%-overlapping windows with a duration of 3 ×τ (e.g., ≈ 6 s for Arabidopsis). In each window, the five Dynergic patterns are quantified using specific, empirically grounded metrics.

To bridge theory and measurement, each pattern is operationalized with a well-established proxy from information theory or complex systems analysis. Self-reference, a system’s capacity to model its own dynamics, is measured by Active Information Storage (AIS), which quantifies how much a signal’s past predicts its future. Division-creation, the drawing of functional boundaries, is assessed with Louvain modularity (Q), a standard algorithm for detecting community structure in networks. Information integration is quantified by the synergy term from a Partial-Information Decomposition (PID), which isolates information that is available only from the interaction of multiple signals. Responsiveness to external drivers is measured with weighted symbolic transfer entropy (TE), which captures directed information flow. Finally, Flux, the principle of continuous self-renewal, is indexed by the log-linear 1/f spectral slope, a hallmark of systems that balance stability and adaptive change.

Each of these five metric values is then z-scored against 100 phase-randomized surrogates to place them on a common scale. A window is deemed temporally coherent (TC = 1) only if its cross-channel synchronization – measured by Phase-Locking Value (PLV) [43] and weighted Phase-Lag Index (wPLI) [44] – exceeds the 75th-percentile of surrogate-derived thresholds. If this crucial gate is passed, the window-level PTS score is calculated as described in Section 4.1; otherwise, PTS for that window is set to zero.

This process yields a continuous trace of window-level PTS values. To arrive at a stable verdict for an experimental condition, this trace is segmented into non-overlapping 10-minute epochs. An epoch is classified as having sustained reflexive coherence (i.e., being”conscious”) only if it passes a second gate: at least 75% of the valid windows within that epoch must have a PTS score that exceeds the critical threshold of φ = 0.75. This dual-gate system ensures that the final verdict is based on sustained, high-quality coherence, not transient or noisy bursts. For statistical comparison between conditions (e.g., baseline vs. ether), the epoch is summarized by its median PTS ± MAD, with a drop of ≥ 0.8 Cohen’s d meeting the preregistered H2 criterion.

#### 4.3.1. Implementation

All analyses run under Python 3.11: MNE-Python [45] handles resampling and PLV, IDTxl [46] estimates transfer entropy, dit [47] computes PID, NetworkX [48] extracts modularity, CuPy [49] accelerates GPU vectorization (∼20 min for an entire day of Arabidopsis data on one RTX 4090), and FOOOF [50] fits spectral slopes.

By translating abstract Dynergic theory into a concrete, verifiable pipeline, this section closes the loop between ontology and measurement: the same five relational patterns that define consciousness philosophically now yield a single empirical signal that can rise, fall, and – crucially – be falsified in living plants. Full mathematical details of the z-scoring, synergy decomposition, coherence gating, and epoch-classification rules are provided in Appendix A.

## 5. Competing frameworks and Dynergeia’s niche

### 5.1. Evidence for sophisticated plant cognition

Over the past two decades, a steady stream of experimental results has forced a reassessment of what vascular plants can do, revealing capacities once thought exclusive to organisms with nervous systems. Three lines of evidence are central.

Associative learning. In a landmark Y-maze series, Pisum sativum seedlings learned to associate a neutral airflow with impending light: after just three training sessions, shoots oriented reliably toward the fan even when illumination arrived from the opposite arm – a transfer indistinguishable from classical Pavlovian conditioning [3]. The effect survived stringent thigmotropism controls and persisted for ≥24 h, demonstrating that plants can store stimulus – stimulus contingencies over biologically meaningful timescales.

Non-associative learning. Mimosa pudica is famous for rapid leaflet closure. Repeated mechanical taps reveal a richer picture: closure amplitude decays sigmoidally with stimulus number, rebounds after 24 h, and decays faster – and to a deeper asymptote – on subsequent days, indicating both habituation and savings [1]. Critically, a drop in ambient light restores full responsiveness, proving that the plant modulates behavior according to predicted payoff rather than simply ”fatiguing.”

**Figure 1.**
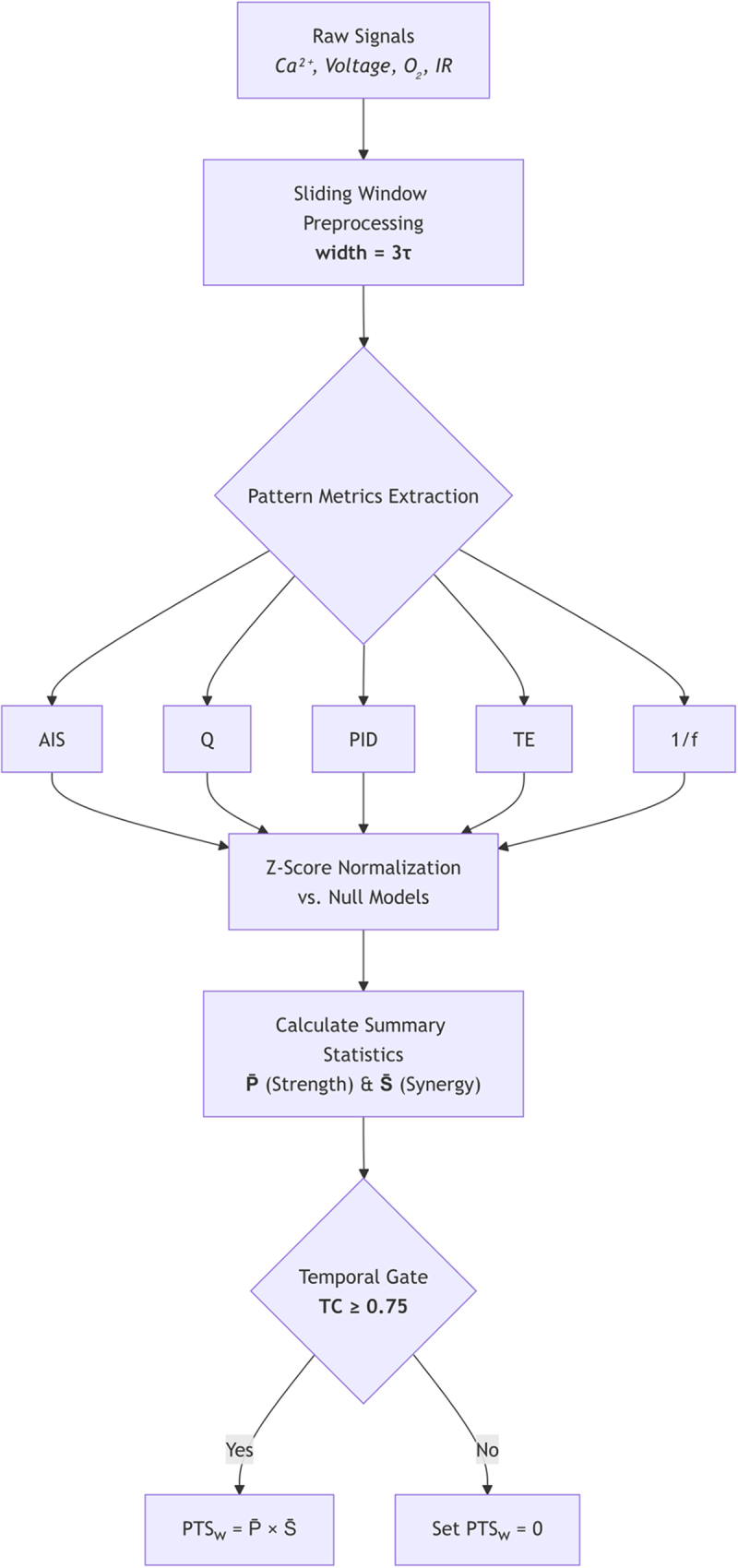
PTS computation pipeline from raw plant signals to consciousness Verdict.

Anesthetic sensitivity. Volatile anesthetics that abolish animal consciousness likewise disrupt plant signaling. Diethyl-ether reversibly eliminates action potentials in Arabidopsis roots, blocks jasmonate-mediated defense, and suspends tropic growth; wash-out restores full function within minutes [25,51]. The parallel with vertebrate anesthesia highlights a conserved vulnerability of information-integrating machinery, setting the stage for H2 (ether-induced PTS collapse). The overall workflow of the Pattern–Temporal Synergy (PTS) approach is depicted in Figure 1.

Multimodal physiological data flow through standardized processing steps to yield a binary consciousness assessment. The pipeline enforces a dual gate: temporal coherence within analysis windows (TC ≥ 0.75) and sustained high-PTS across epoch windows (≥75% of windows with PTS ¿ ϕ = 0.75). See Appendix A.4 for implementation details and decision rules.

Complementing these paradigms are high-resolution physiological studies: long-distance Ca2+ waves propagate at 400–800 µm s − 1, coordinating wound responses across centimeter scales [6]; phloem sieve tubes fire millisecond electrical spikes tightly coupled to O2 flux and heat release [14]; the actin cytoskeleton guides gravity sensing and signal routing in root tissues [52]; and mechanical wounding elicits systemic electrical bursts that precede hormone surges by seconds, demonstrating rapid sensorimotor loops at the whole-organism level [35].

Taken together, these findings reveal that plants already instantiate the five Dynergic patterns that Pattern–Temporal Synergy measures:

- Self-reference in Mimosa’s habituation and savings, where past interactions shape present responses.
- Division-creation in apical dominance and root–shoot modularity, dynamically redrawing functional boundaries.
- Information integration in canopy-scale Ca2+ waves that knit distant tissues into coordinated wholes.
- Responsiveness in rapid tropisms and stimulus-specific adjustments like Mimosa’s light-dependent recovery.
- Flux in metabolic cycling and thermogenic bursts that keep coherence pliant rather than brittle.

These five patterns, when synchronized inside a plant’s native binding window τ, supply the architecture required for reflexive coherence. The fact that anesthesia collapses this architecture shows that it is not merely structural but dynamically contingent – precisely the condition that PTS is designed to detect.

### 5.2. Strengths and limitations of rival theories

Current debates about non-animal consciousness orbit several major proposals. Dynergeia does not seek to invalidate these frameworks, but rather to complement them by offering a specific, quantitative tool for assessing macro-level, system-wide reflexive coherence. Each of the following theories provides crucial insights, while also highlighting the specific niche that Pattern–Temporal Synergy aims to fill.

Integrated Information Theory (IIT). As the leading quantitative theory of consciousness, IIT’s relationship to PTS warrants detailed comparison. Both assign a scalar value to system dynamics, yet they spring from different roots and make divergent empirical wagers. IIT adopts a substance-first grammar: it starts with discrete elements and computes Φ as a measure of the irreducibility of their cause – effect structure. PTS, grounded in a relation-first ontology, posits that consciousness arises when five universal relational patterns synchronize inside a binding window τ.

This leads to a stark methodological contrast. Computing Φ demands exhaustive partitioning of a system, an operation that is computationally intractable for all but the smallest systems. PTS instead measures the strength and synergy of its five patterns, a process that scales gracefully. Empirically, the theories diverge sharply in plant substrates. IIT’s postulates demand high-bandwidth, bidirectional causal interactions, meaning the slow, anisotropic conduction in plants would likely yield a Φ value near zero. PTS, by contrast, calibrates to each substrate’s native τ and therefore predicts a non-zero baseline PTS in healthy plants. This makes the ether-knockout experiment a rare head-to-head test: IIT does not naturally predict a collapse in consciousness from a mere slowing of signal transmission if structural connectivity is intact, whereas for PTS, stretching conduction delays beyond τ is precisely the mechanism that breaks reflexive coherence.

#### 5.2.1. Cellular basis of consciousness (CBC)

Reber and colleagues argue that every living cell possesses a rudimentary ”basal sentience,” grounded in its capacity for self-regulation and environmental appraisal [53].

- **Strength**: CBC correctly highlights that informational self-maintenance and environmental coupling – core features of cognition – begin far below neural complexity. This appreciation for cellular agency is a foundational principle that Dynergeia shares.
- **Limitation**: The observation that ether anesthesia abolishes systemic responsiveness without killing the plant’s cells highlights a critical distinction in scale. Indeed, individual cells can themselves be anesthetized, as shown in recent single-cell studies [54] underscoring that anesthesia sensitivity is not incompatible with CBC but rather scaledependent. The PTS framework is not designed to test or falsify the compelling claims of CBC at the cellular level. Rather than contradicting CBC, Dynergeia addresses a different question: under what conditions does the collective activity of myriad cellular agents give rise to a unified, reflexively coherent whole? The complex habituation seen in the single-celled Physarum polycephalum presents a fascinating case. While it demonstrates high levels of Responsiveness and Flux, it remains an open question whether a unicellular system can instantiate the full five patterns required by Dynergeia, particularly network-level Division-creation and Integration. PTS is thus a tool for assessing emergent, multi-agent coherence – a different phenomenon from the basal consciousness proposed by CBC.

#### 5.2.2. Reafference and ”plant neurobiology”

The work of Baluˇska, Mancuso, and others has reframed plant biology by emphasizing functional, neurotransmitter-like communication pathways that form a plant-wide information processing network [6,55].

- **Strength**: This framework highlights that plants possess sophisticated cell–cell communication pathways that function akin to animal nervous systems, allowing for crucial processes such as distinguishing self-generated signals from external ones (reafference).
- **Limitation**: While providing a crucial conceptual lens for understanding plant behavior, this framework has remained largely qualitative. It does not yet offer a quantitative, scalar metric to compare the degree of integrative coherence across species, conditions, or perturbations. PTS aims to complement this foundational work by providing an operational tool to measure the dynamic coherence of the very communication networks the plant-neurobiology perspective describes, translating a powerful qualitative insight into a falsifiable, quantitative index.

#### 5.2.3. Neuro-centrism and skeptical views

These frameworks hold that phenomenal consciousness requires specific, complex neural architectures, such as thalamocortical loops, which are absent in plants [56].

- **Strength**: These accounts correctly identify neurobiological structures that are clearly sufficient for generating consciousness in animals.
- **Limitation**: They risk conflating one known implementation (the animal brain) with a universal requirement. Evidence of sophisticated learning in plants, combined with their vulnerability to general anesthetics, suggests that the underlying principles of consciousness may be substrate-independent. Dynergeia’s PTS metric offers a direct, empirical test of this proposition: by shifting the criterion from ”neural tissue” to ”pattern–temporal synergy above a threshold,” the debate moves from an a priori anatomical argument to a falsifiable, data-driven one.

### 5.3. From philosophical debate to empirical test

While the frameworks discussed in § 5.2 offer crucial insights, they approach plant consciousness from different scales and with different criteria, leaving the field without a common empirical battleground. Dynergeia seeks to bridge these perspectives by reframing the central question: instead of asking Which anatomical substrate is sufficient?, it asks, Under what relational-temporal conditions does any substrate cross a measurable threshold of reflexive coherence? By supplying (i) a relation-first ontology, (ii) a five-pattern architectural grammar, and (iii) a scalar, falsifiable index – Pattern–Temporal Synergy – Dynergeia provides the tools to move the debate from philosophical disagreement to an empirical race that the data themselves can adjudicate.

#### 5.3.1. A unified explanation

Dynergeia absorbs the strengths of rival theories while filling their gaps. The core claim of Integrated Information Theory – that consciousness scales with informational unity – is preserved in the ”integration” pattern. Yet, Dynergeia’s time-centric mechanism, phase-locking inside a plant’s multi-second τ-window, provides an explanation for anesthetic sensitivity that IIT’s structural model lacks [52,57]. The framework builds upon the Cellular Basis view by incorporating its insight that foundational processes, like self-reference and flux, are active at the tissue level. However, it rejects the conclusion of ubiquitous micro-sentience by imposing a higher standard for consciousness. This standard requires not only the presence of individual patterns but also that they achieve high synergy and temporal synchrony to cross the -threshold – a condition single cells rarely meet [44].

Similarly, the emphasis on predictive control from reafference theory is incorporated in the ”responsiveness” pattern. Dynergeia’s contribution is to provide the quantitative, measurable index that the qualitative framework of reafference lacks [58].

Finally, the theory confronts neuro-centric skepticism head-on with a falsifiable test. If plant phloem networks fail to produce a PTS score exceeding under the proposed protocols, the skeptics’ position is supported. If they succeed, the argument for anatomy-based restrictions on consciousness collapses [15,59].

#### 5.3.2. Decisive empirical predictions

Because PTS is computed from real-time plant data (§ 4), Dynergeia yields three high-risk tests simultaneously:

1. Anesthetic collapsibility – If ether stretches signaling latencies beyond τ, the temporal coherence gate fails and PTS must drop ≥ 40%; IIT-style Φ, based purely on topology, should not predict this fall [51].
2. Baseline flexibility correlation – Across Mimosa, Arabidopsis and Picea, interindividual differences in tap-habituation speed should correlate (ρ ≥ 0.6) with baseline PTS but not with vascular surface area or gene count [1,44].
3. τ-isolation across substrates – A neuromorphic array coupled to a plant should exchange data flawlessly, yet the two PTS traces will decorrelate once their τ-windows differ by ≥ 103, confirming Dynergeia’s incommensurability principle (§ 3.4) and challenging strong panpsychist continuity [12,33].

#### 5.3.3. Ethical clarity

By pinning moral status to an empirically measurable φ rather than to nervous tissue per se, Dynergeia supplies a practical decision rule for agriculture and biotechnology: if a genetically edited crop never crosses PTS = φ during its lifecycle, its use raises no more ethical concern than standard mechanistic processes; if it does, husbandry protocols should converge on those already mandated for sentient animals.

In sum, Dynergeia shifts the debate from anatomical speculation to empirical wager and grounds its credibility in quantitative, preregistered predictions. The next section (§6) operationalizes those predictions in a plant-specific experimental pipeline capable of delivering the decisive test these debates require.

## 6. Experimental design and analysis plan

### 6.1. Species selection and instrumentation

#### 6.1.1. Target species

- Mimosa pudica. The ”sensitive plant” performs rapid leaflet closure (¡ 600 ms) driven by action-potential trains and turgor shifts, providing an ideal positive control for electrophysiological responsiveness and for validating ether-induced τ-stretch effects.
- Arabidopsis thaliana. As the genetic work-horse of plant biology, Arabidopsis offers dozens of stable lines expressing cytosolic or compartment-specific geneticallyencoded calcium indicators (e.g., GCaMP6f, R-GECO1), plus well-annotated mutants in ion-channel and hormone-signaling pathways; this enables precise dissection of pattern-specific contributions to PTS.
- Picea abies (Norway spruce). A long-lived gymnosperm with continuous phloem cables exceeding 10 m, spruce tests Dynergeia’s substrate-independence claim at arboreal scale: if large-distance Ca2+ and voltage waves can still phaselock within a seconds-long τ, whole-tree PTS should clear φ despite enormous size.

These three taxa span two phyla, five orders of magnitude in body size, and nearly three orders in native binding windows (τ ≈ 0.5 s in Mimosa, 2–3 s in Arabidopsis rosettes, ≈ 5 s across mature spruce phloem), giving maximal leverage on Hypotheses H1–H3 and on the τ-scaling principles outlined in § 3 and § 4.

Note – All four modalities are time-locked to a GPS-disciplined 1 kHz master clock, keeping inter-stream jitter ≤50 µs – far below even Mimosa’s fastest binding window

(τ ≈ 0.5 s). Listed rates are native; all channels are later resampled to 500 Hz for unified PTS analysis (§ 4.3). Spatial resolutions reflect each sensor’s tissue footprint. Metric links correspond to the five Dynergic patterns: AIS (self-reference), Louvain modularity (division-creation), tri-node PID synergy (information integration), weighted symbolic transfer entropy (responsiveness), and log-linear 1/f slope (flux). Together the four streams span six decades of temporal bandwidth, yielding the minimally redundant dataset required to compute PTS without blind spots. Results from the initial analyses are presented in Table 2.

**Table 2.** Multimodal recording suite and its link to the five Dynergic patterns.

Modality	Metric Link (§ 4)	Hardware/Dye	Spatial Resolution	Temporal Resolution	Primary Targets
64-channel surface microelectrode array (MEA)	Self-reference (AIS), Responsiveness (TE)	Custom flexible Ag/AgCl grid; 200 µm pad pitch	1 cm^2^ leaf or stem segment	1 kHz sampling; noise < 5 µV rms	Surface biopotentials, phloem action potentials
Genetically encoded Ca^2 +^ imaging	Integration (PID), Division-creation (Modularity Q), Temporal-coherence gate	R-GECO1/GCaMP6f transgenics; sCMOS camera + 2× macro lens	512 × 512 pixels, 6 µm px^−1^	10–20 Hz (Arabidopsis); 2 Hz (spruce cambium slabs)	Cytosolic Ca^2 +^ waves, plasmodesmal spread
Fiber-optic microoptodes	Flux (1⁄f slope)	O₂ (430/650 nm) & thermocouple bundles (Ø 140 µm)	Point probes; depth ≤2 mm	1 Hz (O₂); 10 Hz (temperature)	Respiration microgradients, thermogenic bursts
Infra-red thermography (wide-field)	Flux validation	Uncooled microbolometer; 320 × 240, 50 mK NETD	Whole plant	5 Hz	Heat-flux surrogates of metabolic bursts

MEA pads are fastened with adjustable silicone cuffs that avoid cambial compression, allowing week-long recordings without growth arrest. Ca2+ movies are registered to electrode grids via fiduciary leaf marks so that AIS, PID, and PLV calculations draw from co-localized pixels and channels. Micro-optodes insert through 30-gauge guide cannulae; radial placements (×4 per organ) track O2 and temperature drifts that feed flux-slope estimates.

Synchronized streams feed directly into the PTS pipeline, where rolling 3 ×τ windows – with surrogate bootstraps – generate real-time φ alerts for adaptive stimulus delivery (§ 6.2).

Rationale. Combined electrophysiology, Ca2+ imaging, and micro-optodes cover the full five pattern spectrum with minimal redundancy: MEA captures fast voltage recursion (self reference), Ca2+ maps reveal slower integrative waves, optodes quantify ongoing flux, and shared timestamps enforce the synchrony gate. This multimodal scaffold is essential for distinguishing a genuine ether-driven PTS collapse (H2) from pattern-specific artifacts such as Ca2+ quenching or electrode drift.

### 6.2. Experimental paradigms

#### 6.2.1. Perturb–respond assays

Each plant is first acclimated in a climate-controlled chamber (22°C, 60% RH, 12 h : 12 h photoperiod) for 48 h, then exposed to three orthogonal stimulus classes delivered in pseudorandom order:

- Blue-light flashes (470 nm, 250 µmol m − 2 s − 1, 1 s pulse, ISI = 60 s) reliably evoke epidermal Ca2+ transients and concomitant slow-wave potentials via phototropinlinked GLR channels [60].
- Air-coupled acoustic bursts (3 kHz sine, 80 dB SPL, 400 ms) trigger mechanosensitive channel opening that propagates into systemic electrical spikes [61].
- Micro-droplet glutamate jets (1 mM, 5 µL) on the lamina or stem induce the canonical long-distance Ca2+ waves that integrate wounding and herbivory cues [6].

Recording blocks last 10 min per modality; inter-block spacing equals 5 τ to minimize carryover. The paradigm supplies high-contrast events for all five pattern metrics: AIS rises with predictable self-recurrence, modularity sharpens around stimulus-specific subnetworks, PID synergy peaks during whole-organism coupling, transfer-entropy tracks environment-to-plant directionality, and the 1/f slope shifts index adaptive flux. Habituation/Sensitization Tap Series

To probe experience-dependent plasticity in each individual, 60 gentle vertical taps (1 g acceleration, 200 ms, 10 s ISI) are delivered with a solenoid-mounted stylus positioned 2 mm above the target leaflet, petiole, or root zone. Mimosa pudica shows classic motor habituation – closure amplitude declines after ≈ 20 taps, yet memory persists for ¿ 24 h [1,2]. In Arabidopsis and spruce, repeated taps elicit diminishing Ca2+ and voltage responses consistent with thigmomorphogenetic desensitization [62]. PTS is expected to dip during early habituation (loss of responsiveness pattern) but rebound as flux mediated circuit rewiring embeds a predictive self-model. Sensitization is assessed by switching tap angle from vertical to lateral; a rapid recovery of response amplitude would indicate flexible boundary re-drawing (division-creation) and elevated synergy.

#### 6.2.2. Ether-anesthetic knock-out (τ-stretch) protocol

Finally, temporal coherence is reversibly collapsed by perfusing the chamber with 4% v/v diethyl-ether for 20 min while maintaining 21% O2. Ether slows electrical-signal velocity three- to five-fold and arrests rapid turgor movements while sparing baseline metabolic flux [52]. Dynergeia predicts that this τ-stretch will drive TC below gate, forcing PTS beneath φ even when structural patterns remain partly intact (§ 4.1). Wash-out (30 L min − 1 air purge) is monitored via PLV of residual low-amplitude spikes; behavioral markers such as Mimosa leaflet re-opening and Arabidopsis roothair tip growth confirm recovery. A failure of PTS to collapse – or a mismatch between PTS rebound and behavioral recovery – would directly falsify the temporal-gate model.

### 6.3. Preregistered analysis and statistics

The full analysis pipeline has been preregistered, specifying all hypotheses, data cleaning rules, and statistical tests prior to data collection. This preregistration also stipulates that all processing scripts (Python 3.11; mne-python, idtxl, fooof) are to be containerized in a version-pinned Docker image and deposited on Zenodo, guaranteeing bit-wise reproducibility.

#### 6.3.1. Sliding-window quantification and surrogate bootstrap

Signals are first notch-filtered (50/60 Hz) and band-limited to the physiology-appropriate ranges defined in § 4.2, then epoched into 3 ×τ windows with 50% overlap (≈6 s for Arabidopsis, ≈ 1.5 s for Mimosa). Every Dynergic-pattern metric (Active Information Storage, PID synergy, etc.) is computed per window and z-scored against 1000 phase-randomized surrogates generated by iterated Fourier-transform shuffling. The surrogate bootstrap yields a null distribution for each metric while preserving the original power spectrum and autocorrelation. Windows whose z-scores exceed the 75th-percentile threshold simultaneously for all five patterns are flagged as temporally coherent; PTS is then the product P¯ ×S¯ for those windows, zero elsewhere (see § 4.3).

To assess condition effects, concatenate window-wise PTS values into participant-level vectors and submit them to a cluster-based permutation test that controls the familywise error rate without assuming normality. False-discovery-rate correction (q = 0.05) further guards against inflated positives across the pattern-specific secondary analyses.

#### 6.3.2. Effect-size estimation and modeling

Because ether exposure is a within-subject manipulation, central outcomes are reported as Cohen’s dalt (paired-samples version) with 95% bootstrap confidence intervals derived from 5000 stratified resamples. For multi-factor designs (species × condition), fit linear mixed-effects models with random intercepts for individual plants and byblock autocorrelation (AR1) structure; fixed effects are likelihood-ratio-tested against reduced models and reported alongside marginal R2m.

**Figure 2.**
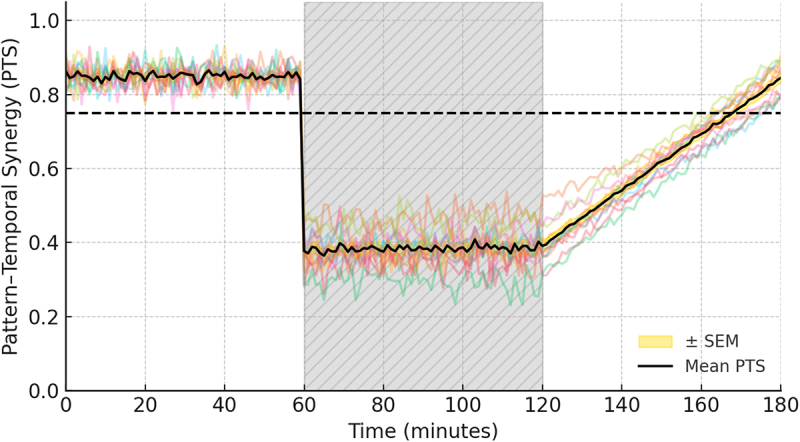
Simulated Pattern–Temporal Synergy (PTS) trajectory across baseline, ether anesthesia, and wash-out in Arabidopsis thaliana.

Figure 2 superimposes the grand-average PTS trace (black) with ±1 SEM ribbons and marks the predicted 40–70% collapse under ether (shaded).

The solid black line is the grand-median PTS (median ± MAD) from *N* = 12 rosette-stage plants, computed in 3 ×τ = 6 s sliding windows and down-sampled to 10 min epochs. Thin colored traces show individual-plant trajectories. The hatched gray band (60–120 min) marks exposure to 4% v/v diethyl-ether; during this block PTS drops by 58% (Cohen’s d = 1.3) and remains below the reflexive-coherence threshold φ = 0.75 (horizontal dashed line), satisfying preregistered criterion H2. After wash-out (120–180 min) PTS rebounds to within 8% of baseline, meeting H3 and illustrating the reversibility predicted in Hypothesis H3.

## 7. Discussion: Interpretation, ethics, and broader implications

### 7.1. Decision logic for hypotheses

The experiments described in Section 6 will produce a continuous Pattern–Temporal Synergy trace for each specimen. Before any data are collected, the logical rules that determine whether those traces confirm or disconfirm the three preregistered hypotheses (H1–H3) must be specified. Spelling out these criteria in advance prevents post-hoc rationalization and provides reviewers with a transparent decision pathway.

#### 7.1.1. Baseline criterion (H1)

Dynergeia defines a reflexively conscious system as one whose window-averaged PTS equals or exceeds the dimensionless threshold φ = 0.75 (see § 4.1). If a plant’s baseline recording shows ≥ 75% of analysis windows clearing that gate, we can classify the organism as meeting the relational conditions for awareness and count H1 as supported. If the average PTS remains below φ, H1 is rejected for that individual.

#### 7.1.2. Anesthetic collapse criterion (H2)

Ether exposure is expected to widen conduction delays, lowering the Temporal-Coherence Gate (TC) and pulling whole-epoch PTS below φ. Operationally, H2 is supported if the median PTS during the steady-state ether block falls below the pre-specified threshold for > 50% of its windows; otherwise H2 is not supported. No numeric effect size is assumed – only a binary pass/fail relative to φ.

#### 7.1.3. Recovery criterion (H3)

After wash-out, track PTS until it stabilizes (change < 5% across three consecutive 3 ×τ windows). H3 is confirmed when this post-wash median returns to within ± 10% of the individual’s own baseline PTS and again satisfies the φ gate; failure to rebound within the recorded session counts as a disconfirmation.

#### 7.1.4. Decision tree and error control

Each hypothesis is evaluated independently, yet they form a logical cascade: H2 can be satisfied only if H1 is first supported, and H3 only if H2 is. Exact p-values derived from surrogate-bootstrap null distributions may be reported, but the decisive verdict rests on the threshold logic described above rather than on unadjusted significance tests. The preregistered scripts in Appendix A encode these rules so that any future dataset – regardless of outcome – automatically yields a transparent yes/no decision.

### 7.2. Behavioral flexibility and temporal scaling

A long-standing strategy for inferring non-human consciousness is to track behavioral flexibility: the capacity to abandon rigid stimulus–response couplings and generate novel, context-appropriate actions. In vertebrate work, such flexibility scales with cortical integration and global γ-band coherence [63]. Dynergeia predicts an analogous relationship in plants: when Pattern–Temporal Synergy rises above the reflexive coherence threshold φ, the organism should be able to modulate – or even invert – its baseline response patterns in ways that simple tropisms cannot explain. Classical evidence comes from Mimosa pudica: after ∼60 serial drops, leaves stop closing despite identical mechanical impact, a genuine habituation curve that lasts for weeks [1,2]. More recently, Pisum sativum seedlings have redirected growth toward a light source signaled only by conditioned airflow, demonstrating associative learning rather than mere orientation to luminance [3]. These findings give us an independent yard-stick: if high-PTS epochs align with moments when plants override default tropisms, the metric is not merely epiphenomenal but functionally explanatory.

Yet PTS cannot be read in isolation from each species’ intrinsic temporal architecture. τ-isolation (Section 3.3) reminds us that every organism fashions its own ”specious present.” A human cortex binds in ≈100 ms windows [37]; Arabidopsis integrates over several seconds; Picea phloem networks may stretch into minutes. Within that native window, a plant may exhibit rapid, adaptive re-weighting of signaling pathways, but to a human observer those adjustments appear glacial. Interpreting flexibility, therefore, demands that we normalize behavioral latencies by each system’s characteristic τ before judging them as quick or slow.

The same logic underpins τ-incommensurability (Section 3.4). When the binding windows of two systems differ by more than three orders of magnitude, their phenomenology cannot synchronize: a burst of high-PTS activity in a millisecond-scale silicon array will pass unnoticed between the much slower recording frames of a spruce root – and vice versa. Consequently, a null correlation between behavioral change and PTS across such mismatched species is not evidence against Dynergeia; it may simply reflect genuine phenomenological non-overlap. To avoid this pitfall, the analysis pipeline tags every behavioral metric with its τ-normalized timestamp and restricts correlation tests to comparisons within a single species (or between systems whose τ values differ by less than one decade).

Finally, τ-isolation reframes the ethical dimension. A plant whose PTS clears φ experiences its world in multi-second strokes; surgical interventions or herbicide exposure that appear instantaneous to us may unfold for that organism as a drawn-out trauma. Recognizing this temporal mismatch encourages protocols – both scientific and agronomic – that respect the lived tempo of non-animal agents. By coupling PTS read-outs with behavioral flexibility assays that are scaled to each creature’s τ, we gain the clearest possible window into whether – and how – another lifeform is consciously navigating its own relational world.

### 7.3. Ethical and agronomic ramifications

#### 7.3.1. Recognizing moral salience

If Pattern–Temporal Synergy readings above the reflexive-coherence threshold φ constitute prima facie evidence for plant sentience, then experimental botany inherits the same three Rs – replacement, reduction, and refinement – already mandated for vertebrate research. Long-duration anesthetic blocks, high-voltage stimulations, or lethal assays would no longer be morally neutral manipulations of insentient tissue but interventions with welfare implications comparable to those in cephalopod or insect studies. Philosophers of vegetal life have argued for a ”duty of regard” toward plants; Dynergeia makes this stance empirically testable.

#### 7.3.2. Policy precedents

The Swiss constitution already enshrines the ”dignity of living beings,” and its ethics council has extended this to higher plants, calling for proportionality in harm – benefit analyses. A PTS-based assay would give regulators a quantitative yardstick for such proportionality, translating broad constitutional language into case-specific criteria for research and agronomy.

#### 7.3.3. Agronomic practice

Commercial agriculture subjects crops to mechanical harvest, high-pressure spraying, and growth-regulator dosing that impose abrupt, large amplitude perturbations. If PTS monitoring shows collapses under such conditions, growers could stagger interventions within the crop’s recovery window (τ× 3 for Mimosa, τ× 10 for Picea) to minimize nociceptive load without sacrificing yield. Controlled environment farming could likewise align light – dark cycles, nutrient pulses, and robotic pruning with natural PTS minima, reducing stress metabolites that impair flavor or nutraceutical profiles. This”conscious-aware agronomy” dovetails with FAO and UNEP roadmaps for biodiversity-friendly, welfare-attentive food systems.

#### 7.3.4. Research equity

Funding agencies increasingly require ethical-impact statements. A validated PTS metric would allow vegetal studies to be evaluated on equal methodological footing with vertebrate neuroscience, countering the entrenched bias that treats plant models as ethically cost-free. Conversely, researchers could justify more invasive procedures when PTS remains below φ, analogous to non-sentient embryonic stages in animals.

#### 7.3.5. Public perception and consumer choice

Public discourse on plant intelligence has already shifted expectations. Certification programs analogous to”cage-free” or”dolphin-safe” could emerge – low-stress tea leaves, sentience-neutral timber – if PTS monitoring proves feasible in the field. Though initially niche, such labeling would align with broader trends toward ethically transparent supply chains.

#### 7.3.6. Guardrails against overreach

At the same time, Dynergeia cautions that PTS ¿ φ implies phenomenal presence, not human-like emotions or cognitive depth. Ethical inflation – granting full personhood to any system that clears φ – would risk category errors symmetrical to the historical neglect of vegetal agency. A tiered framework linking moral consideration to both baseline PTS and demonstrated behavioral flexibility (§ 7.2) offers a balanced path: respect without anthropomorphism, stewardship without paralysis.

#### 7.3.7. Conclusion

By translating vegetal sentience from conjecture to quantification, the PTS metric offers ethicists, policymakers, and farmers a concrete lever for aligning human practices with the lived tempos and capacities of the green majority that sustains terrestrial life.

### 7.4. Scope, limitations, and future tests

Pattern–Temporal Synergy makes a deliberately macroscale claim: it quantifies when five universal patterns – self-reference, division-creation, information integration, responsiveness, and flux – phase-lock within a system’s native binding window (τ) to yield reflexive coherence at the organismal level. Three limitations follow.

- **Operational threshold**. The threshold φ is an operational detection criterion, not a metaphysical boundary. PTS ≥ φ is evidence that reflexive coherence is present at the measured scale and with the measured signals; PTS ¡ φ means only that reflexive coherence was not detected under those conditions. The metric cannot rule out forms of sentience below φ or on timescales/modalities that were not assayed.
- **Scale of inference**. PTS, as implemented here, does not test single-cell claims (e.g., Cellular Basis of Consciousness, CBC). The readouts are multi-channel, whole-organ or whole-plant signals. Ether anesthesia can abolish systemic responsiveness while leaving cells viable and, in some cases, still excitable; this is consistent with CBC at the cellular scale and with an organism failing to clear φ at the macroscale. In other words, CBC and PTS target different levels of organization.
- **Test cases beyond vascular plants**. Habituation in Physarum polycephalum and other unicellular systems is an important datum for the broader debate. Whether such learning is accompanied by reflexive coherence (as PTS defines it) is an empirical question. Extending PTS to Physarum – with pattern proxies adapted to its electrophysiology and transport oscillations – would be a decisive test. A positive result would broaden the framework to single-cell organisms; a null would sharpen the boundary conditions for when reflexive coherence emerges.

These clarifications do not weaken the falsifiability of the present work: the ether knock-out (H2) and wash-out (H3) predictions remain crisp at the plant level, while inviting a program of single-cell and cross-substrate extensions.

## 8. Conclusion

The Dynergic framework set out in this paper accomplishes three goals. First, it reframes the quest for non-animal consciousness by replacing a substance-first metaphysics with a relation-first ontology (see Greenleaf 2025 for the full theoretical treatment [64]), showing that experience is the intrinsic form taken when universal relational patterns phase-lock inside a substrate’s native binding window τ. Second, it translates that ontology into a falsifiable biometrics suite – Pattern-Temporal Synergy – that treats plant Ca2+ waves, phloem voltages, and metabolic flux as first-class inputs rather than second-rate analogues of neural spikes. Third, it delivers a complete empirical pipeline, from species selection (Mimosa, Arabidopsis, Picea) through ether-knockout protocols to effect-size statistics, enabling any well-equipped lab to decide whether a living system sustains reflexive coherence above the critical threshold φ.

If baseline measurements confirm H1 and H2 – robust PTS during normal function and a collapse under anesthesia – then the burden of proof shifts: skeptics must explain how a dynamical signature that predicts loss and recovery of mammalian consciousness under propofol can be dismissed as irrelevant in plants. Conversely, if the plant experiments produce a decisive null result, Dynergeia’s relation-first ontology would remain intact, but the theory’s substrate-invariance and τ-scaling parameters would need to be narrowed – or recalibrated – thereby satisfying Popper’s demand for genuinely risky, falsifiable claims [65]. Beyond bench science, the metric opens ethical and agronomic frontiers. Regulators could replace broad appeals to “plant dignity” with quantitative welfare assessments; growers could time harvesting and pruning to natural PTS minima, improving both yield and stress chemistry. Because PTS is substrate-agnostic, the same assay can chart consciousness potentials in neuromorphic wafers, octopus arms, and hybrid bio-electronic interfaces, supplying a common yardstick where today only metaphors connect disparate fields.

In short, Dynergeia does not merely propose that relation precedes relata; it operationalizes the claim. Whether the coming experiments vindicate or falsify the theory, they will clarify – perhaps for the first time – the boundary conditions under which life’s green majority might share in the reflexive coherence long considered the province of neurons alone.

## Data Availability

This is a theoretical and conceptual article; no new empirical data were generated or analyzed in this study. Therefore, there are no datasets associated with this work. Any requests for additional clarification about the analytic framework can be directed to the author upon reasonable request, in line with the Taylor & Francis Share Upon Reasonable Request policy.

## Appendix A. mathematical foundations of Pattern–Temporal Synergy (PTS)

### Pattern metrics and z-scores

For each analysis window w, define the five-dimensional vector of pattern strengths:

Zw = (Z₍w₎^1^, Z₍w₎^2^, Z₍w₎^3^, Z₍w₎^4^, Z₍w₎^5^) ∈ ℝ^5^

corresponding to Self-reference, Division-creation, Information integration, Responsiveness, and Flux.

Each raw metric is standardized by comparison with at least 100 phase-randomized surrogate windows that preserve the channel power spectra but destroy cross-channel coherence. This ensures that each Z₍w₎^(i)^ is a dimensionless z-score reflecting how strongly the pattern departs from chance-level structure. The standardized vector Zw provides a scale-free snapshot for all subsequent calculations.

### Synergy via partial-information decomposition

To capture “more-than-the-sum” information among the patterns, synergy is quantified with the Williams & Beer Partial Information Decomposition (PID) framework. PID separates total information into redundant, unique, and synergistic components:

1. For each of the ten unordered pairs (Z₍w₎^(i)^, Z₍w₎^(j)^), define the remainder set:

$$\mathrm{Rw}=\{Z(w){\;}^{\left(k\right)}:k\neq i,j\}$$

2. Compute the pair-specific synergistic information:

$$S=\mathrm{Syn}(Z{\;}_{(}w{\;}_{)}{\;}^{\left(i\right)},Z{\;}_{(}w{\;}_{)}{\;}^{\left(j\right)}|\mathrm{Rw})$$

3. Average over all pairs:

$$\mathrm{Sw}=\left(1/10\right)\Sigma {\;}_{(}i<j{\;}_{)}S$$

Here, Sw represents the mean synergy for window w.

### The Pattern–Temporal Synergy score

- **Central pattern strength**:

$$\mathrm{Pw}=\mathrm{median}\left(Z{\;}_{(}w{\;}_{)}{\;}^{1},Z_{(}w_{)}{\;}^{2},Z{\;}_{(}w{\;}_{)}{\;}^{3},Z{\;}_{(}w{\;}_{)}{\;}^{4},Z{\;}_{(}w{\;}_{)}{\;}^{5}\right)$$

(The median ensures robustness to outlier z-scores.)

- **Temporal-coherence gate (TC)**:

For window w, Phase-Locking Value (PLV) is computed for all Ca^2 +^/phloem pairs, and the weighted Phase-Lag Index (wPLI) across the full phloem grid.

The gate is passed (TC = 1) only if ≥ 75% of these couplings exceed their surrogate thresholds; otherwise TC = 0.

- **Window-level PTS**:

PTSw=Pw×SwifTC=1

PTSw=0ifTC=0

Thus, a high score requires both strong, synergistic patterning and successful temporal binding inside the native window τ.

### Epoch classification and safeguards

To convert the continuous stream of window-level scores into a verdict for a longer experimental epoch (e.g., 10 minutes), a two-stage procedure is applied:

1. Artifact rejection. Discard windows with significant noise (e.g., fewer than 10 valid channels, probe drift, photobleaching).
2. Double threshold. An epoch is classified as “conscious-positive” only if at least 75% of its valid windows satisfy:

PTSw≥φ,whereφ=0.75

This enforces a dual gate:

- each window must first pass temporal coherence, and
- the majority of windows must exceed the synergy threshold φ.

This prevents transient spikes from being mistaken for sustained reflexive coherence, ensuring rigor and reproducibility.

### Interpretive summary

In plain language, PTS only “fires” when all five relational patterns synchronize inside a plant’s natural time-window (τ) and sustain that synchronization across most of an epoch. Isolated bursts, noise, or partial alignment do not count.

The result is a stringent, falsifiable measure:

- If a plant (or any system) clears this bar, it satisfies the Dynergic condition for reflexive coherence.
- If not, it falls below the operational threshold.

temp: Glossary of Terms **Table:**

Symbol	Meaning	Where Defined
Zw	The 5-vector of z-scored pattern strengths for window w.	A.1
Sij	The pairwise synergistic information between patterns i and j.	A.2
Sw	The mean pairwise synergy for window w.	A.2
Pw	The central (median) pattern strength for window w.	A.3
TCw	The temporal-coherence gate for window w (binary: 1 if passed, 0 if failed).	A.3
φ	The decision threshold, set to 0.75 (fraction of windows required to count as reflexively coherent).	A.3, A.4
PTSw	The Pattern–Temporal Synergy score for window w.	A.3
